# Has retinal gene therapy come of age? From bench to bedside and back to bench

**DOI:** 10.1093/hmg/ddz130

**Published:** 2019-06-25

**Authors:** Ivana Trapani, Alberto Auricchio

**Affiliations:** 1 Telethon Institute of Genetics and Medicine (TIGEM), Pozzuoli, Italy; 2 Medical Genetics, Department of Translational Medicine, Federico II University, Naples, Italy; 3 Department of Advanced Biomedicine, Federico II University, Naples, Italy

## Abstract

Retinal gene therapy has advanced considerably in the past three decades. Initial efforts have been devoted to comprehensively explore and optimize the transduction abilities of gene delivery vectors, define the appropriate intraocular administration routes and obtain evidence of efficacy in animal models of inherited retinal diseases (IRDs). Successful translation in clinical trials of the initial promising proof-of-concept studies led to the important milestone of the first approved product for retinal gene therapy in both US and Europe. The unprecedented clinical development observed during the last decade in the field is however highlighting new challenges that will need to be overcome to bring gene therapy to fruition to a larger patient population within and beyond the realm of IRDs.

## Introduction

**Figure 1:**
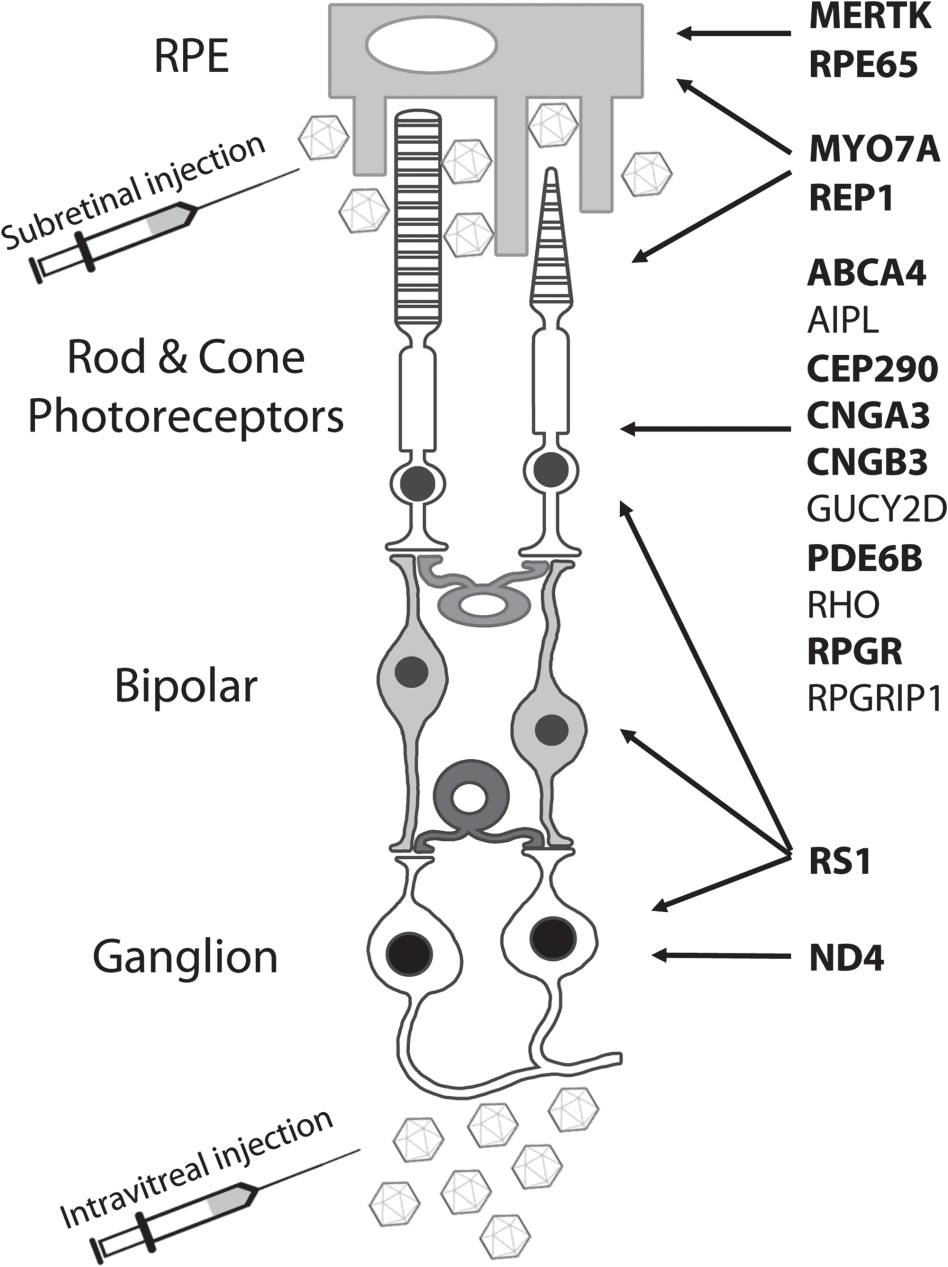
Schematic representation of the retina and pattern of expression of retinal genes. Genes for which gene therapy approaches are in clinical testing are indicated in bold. RPE: retinal pigment epithelium.

With the significant progresses made in the discovery of the genetic bases of many inherited diseases, gene therapy has become a valuable treatment option for many diseases previously considered incurable. While gene therapy was advancing, it became evident that some tissues are more amenable than others to be targeted by gene delivery. Among them is the eye, which offers several advantages. First, due to its small size and accessibility, vectors delivered to the eye can be placed in close proximity of the cell types of interest rather than being administered systemically as it needs to be done to target other organs. This significantly reduces the total amount of vector required to obtain efficacy. Additionally, because of the confined compartmentalized nature of the eye, minimal systemic dissemination of the gene therapy vector occurs, which reduces the risks of the treatment. Further, the eye, and in particular the subretinal space, is relatively immune privileged, thus the exposure to foreign antigens, including viral vectors used for gene therapy, is generally well tolerated and does not elicit potentially damaging immunologic reactions (1,2). Also, visual function and retinal anatomy can be monitored after treatment using noninvasive methods (3) and, since inherited retinal diseases (IRDs) are typically bilateral diseases with significant symmetry (3), one eye can be treated at a time, which allows the fellow untreated eye to act as a control. Lastly, surgical procedures may be adapted to preferentially transduce a particular ocular cell type, with minimal risks to patients undergoing surgery. The administration route is in fact a major determinant of specificity and efficacy of retinal gene delivery. The two most common routes of administration are intravitreal and subretinal injections (4). The intravitreal injection releases the therapeutic agent in the vitreous (Fig. 1) and exposes the anterior retina to transduction. Subretinal injections, alternatively, deliver the vector into a virtual space between the retinal pigment epithelium (RPE) and the photoreceptors (PRs, Fig. 1), inducing a regional and reversible detachment. Despite its greater ease of administration and wider vector distribution, intravitreal delivery has shown, thus far, less success than subretinal at transducing PRs and RPE, which are the main target cells for the treatment of most IRDs (Fig. 1). This most likely occurs because of the presence of several physical barriers between the vitreous and the outer retina (5) and due to the dilution of the therapeutic agent within the vitreous cavity. Thus, subretinal injection is currently considered to be the most efficient route for targeting the outer retina. Yet, subretinal injections, which result in the transient detachment of the RPE from the underlying PRs, should be avoided in retinas rendered fragile by undergoing degenerative processes. Thus, efforts to optimize the safety and efficacy of subretinal and intravitreal delivery, as well as at identifying alternative injection procedures to achieve transduction of the outer retina from the vitreal side (6), are ongoing, as discussed in the following section.

In addition to the choice of an appropriate delivery route, gene therapy success is dependent on the availability of an efficient vector. In fact, a number of vectors (Table 1) were tested in the past 20 years for gene delivery to the mammalian retina, in an attempt to identify those with the highest RPE and PR tropism, ability to transduce the retina from the vitreous and possibility to accommodate large transgenes.

**Table 1:** Features of viral vectors for gene therapy

	Ad	LV	AAV
Family	Adenoviridae	Retroviridae	Parvoviridae
Particle size (nm)	100	80–100	25
Viral genome	dsDNA	ssRNA	ssDNA
Cloning capacity (kb)	**≤**36	≤8	**≤**5
Cell transduction	Dividing and non-dividing	Dividing and non-dividing	Dividing and non-dividing
Integration	No	Yes	No

Ad: adenovirus; LV: lentivirus; AAV: adeno-associated virus; ds: double-stranded; ss: single-stranded.

### The delivery toolbox for the retina

The first gene therapy vector found to target the retina was derived from an adenovirus (Ad) (7,8). While the RPE was efficiently transduced by Ad, PRs required high vector doses for transduction. However, further studies demonstrated that gene transfer to PRs was increased in the newborn retina and in animal models of IRDs at the pre-degenerate state (8), possibly due to reduced physical barriers to penetration by the relatively large Ad particle (Table 1). This data was soon after followed by the first demonstration of successful gene therapy using Ad vectors in the rd1 mouse model of retinal degeneration (9). Meanwhile, new efforts were undertaken to evaluate the retinal transduction profiles by alternative viral vectors. Subretinal administration of lentiviral vectors (LV) derived from the human immunodeficiency virus type 1 resulted, similarly to Ad, in efficient transduction of RPE and in PRs transduction levels, which were higher in newborn than in adult rats (10), and in the rd mouse retina, which presents weaker physical barriers (11). Since then, other LV vectors have been tested in the eye, derived from either the primate HIV-2, simian, bovine, feline immunodeficiency viruses or equine infectious anaemia virus (EIAV) (12). However, despite the great diversity of Ad serotypes and LV pseudotypes tested thus far, PR transduction in animal models beyond the newborn age remains elusive with these vectors. One possible explanation could be that the large size of Ad and LV particles (Table 1) imposes steric constraints that hamper the virus’ diffusion through the thick physical barriers of the adult retina (11,13,14).

The low levels of adult PR transduction obtained with both Ad and LV vectors have established the small adeno-associated viruses (AAVs) as vectors of choice for gene delivery to the retina. Since the first trials in the retina of animal models, indeed, AAVs have demonstrated efficient transduction of both PRs and RPE after subretinal injection (15,16). Additionally, AAVs have a number of advantages including an excellent safety profile (lack of pathogenicity and low immunogenicity) and ability to provide long-lasting transgene expression after a single injection, at least in post-mitotic tissues, as the eye (17). Lastly, being a small virus (Table 1), it can diffuse easily across biological barriers and within the retina. Dozens of different AAV serotypes have been isolated, which have shown unique transduction characteristics (12). The favorable retinal transduction properties of the first AAV vectors boosted further small- and large-animal studies, as well as attempts to isolate or engineer AAV variants with higher transduction levels, different tropism and ability to transduce the retina through the less invasive intravitreal route (17,18). Among the first approaches to develop AAV with improved characteristics was engineering by rational design. This method is based on altering the capsid either *in silico* or *in vitro* based on the knowledge of the structure-function relationship of the virion (17,18). Successful examples of rational design are the tyrosine-mutant capsids, developed to allow escaping of AAV vectors from intracellular proteasome-mediated degradation (19). Notably, these variants have shown increased transduction abilities as well as broader tropism within the neuronal retina and enhanced diffusion across the retina when delivered from the vitreous (20). A major limitation to rational design is, however, the limited knowledge pertaining to AAV cell surface binding, internalization, trafficking, uncoating and gene expression. Therefore, an alternative method to develop novel AAV variants has been pursued, namely directed evolution, in which, similarly to natural evolution, capsids are isolated under selective pressure to yield genetic variants with specific transduction characteristics. Interestingly, AAV2-7m8 was isolated through directed evolution for its ability to transduce the outer retina from the vitreous in small animal models (21). However, intravitreal injection in large animals, such as dogs and non-human primates (NHPs), of both tyrosine mutants as well as AAV2-7m8, fails to reproduce the same levels of outer retina transduction observed in mice (21–23), presumably because of the presence of thicker barriers in larger animals than in mice, which prevents efficient AAV diffusion. More recently, *in silico* methods for capsid engineering have been developed including phylogenetic and statistical modelling of known capsid sequences to predict ancestral AAV capsid sequences (24). Vectors based on ancestral AAV capsid sequences have shown improved transduction abilities (24).

Non-viral gene delivery has also been considered for retinal gene therapy. Non-viral gene delivery offers advantages over viral vectors in terms of both reduced risks of eliciting an immune response and potential ability to deliver larger transgenes. However, while viruses have evolved to efficiently deliver their genetic material into cells, nuclear entry represents a significant obstacle for non-viral gene delivery. Thus, non-viral gene delivery has thus far shown more limited retinal transduction ability than viral vectors (5,12). Both physical and chemical approaches have been explored to increase the efficiency of non-viral gene delivery to the retina, resulting in some degrees of efficacy in animal models of IRDs (12,25). However, concerns regarding both the duration of the effect of the treatment and the low efficiency of PR transduction, especially in large animal models (26), have limited the broad development of non-viral vectors for retinal gene therapy. Within the field of non-viral gene transfer are antisense oligonucleotides (AON), which can be used to target mutant alleles resulting in aberrant splicing. The efficacy of repeated intravitreal deliveries of AON targeting the common CEP290 IVS26 allele that causes Leber congenital amaurosis (LCA) type 10 has been shown in a recent clinical trial (27). Based on this, a pivotal phase II–III trial (NCT03913143) has been approved and started recruiting patients (28). A similar trial that uses AON to treat Usher Syndrome Type 2A has been recently launched (NCT03780257). These will provide significant information on the therapeutic relevance of AON-based approaches in the retina, which however require multiple administrations.

### Clinical trials of gene replacement for IRDs

Given the advantages that the eye offers as a target organ for gene therapy, it has been at the forefront of translational gene therapy. Gene replacement represents an attractive therapeutic option for those IRDs due to loss-of-function mutations, in which delivery of a normal copy of the gene is expected to restore the lost function. This has been proven in dozens of animal models of IRDs, paving the way for translation in successful clinical trials (12,28). Most studies that have moved to the clinic (Table 2) have used AAV, while other viral and non-viral vectors had limited clinical application so far. For a comprehensive review of proof-of-concept studies using these platforms please refer to (12,29).

**Table 2:** Gene therapy clinical trials for retinal diseases (from clinicaltrials.gov as of April 18th 2019)

**Target disease**	**Treatment**	**Sponsor**	**Phase**	**Number (NCT)**
**Achromatopsia**	AAV8-hCARp-hCNGB3	MeiraGTx UK II Ltd	I–II	03001310
	AAV2tYF-PR1.7-hCNGA3	Applied Genetic Technologies Corp	I–II	02935517
	AAV2tYF-PR1.7-hCNGB3	Applied Genetic Technologies Corp	I–II	02599922
	AAV-CNGB3	MeiraGTx UK II Ltd	I–II (LTFU)	03278873
	AAV8-hG1.7p-coCNGA3	MeiraGTx UK II Ltd	I–II	03758404
	AAV8-hCNGA3	STZ eyetrial	I–II	02610582
**Choroideremia**	AAV2-hCHM	Spark Therapeutics	I–II	02341807
	AAV2-REP1	University of Oxford	II	02407678
	AAV2-REP1	University of Miami	II, completed	02553135
	AAV2-REP1	University of Oxford	I–II, completed	01461213
	AAV2-REP1	University of Alberta	I–II, completed	02077361
	AAV2-REP1	STZ eyetrial	II	02671539
	AAV2-REP1	Nightstar Therapeutics	II	03507686
	AAV2-REP1	Nightstar Therapeutics	III	03496012
	AAV2-REP1	Nightstar Therapeutics	LTFU	03584165
**Leber congenital amaurosis 2**	AAV2-hRPE65v2	Spark Therapeutics	I–II (follow on)	01208389
	AAV5-OPTIRPE65	MeiraGTx UK II Ltd	I, II (LTFU)	02946879
	AAV2-hRPE65v2–301	Spark Therapeutics	III	00999609
	AAV2-hRPE65v2–101	Spark Therapeutics	I	00516477
	AAV5-OPTIRPE65	MeiraGTx UK II Ltd	I–II	02781480
	AAV2-hRPE65p-hRPE65	University College, London	I–II, completed	00643747
	AAV2-CB-hRPE65	Applied Genetic Technologies Corp	I–II, completed	00749957
	AAV2-CBSB-hRPE65	University of Pennsylvania	I	00481546
	AAV2-hRPE65	Hadassah Medical Organization	I	00821340
	AAV4-hRPE65	Nantes University Hospital	I–II, completed	01496040
	AAV2-hRPE65v2	Spark Therapeutics	LTFU	03597399
	AAV2-hRPE65v2	Spark Therapeutics	I–III (LTFU)	03602820
**Leber congenital amaurosis 10**	QR-110	ProQR Therapeutics	I–II	03140969
	QR-110	ProQR Therapeutics	II–III	03913143
	QR-110	ProQR Therapeutics	I–II (LTFU)	03913130
	EDIT-101	Allergan	I–II	03872479
**Leber’s hereditary optic neuropathy**	scAAV2-P1ND4v2	University of Miami	I	02161380
	AAV2-ND4 (GS010)	GenSight Biologics	III, completed	02652780
	AAV2-ND4 (GS010)	GenSight Biologics	III	02652767
	AAV2-ND4	Huazhong University of Science and Technology	II–III	03153293
	AAV2-ND4	Huazhong University of Science and Technology	I–II, completed	01267422
	AAV2-ND4 (GS010)	GenSight Biologics	I–II	02064569
	AAV2-ND4 (GS010)	GenSight Biologics	III	03293524
	AAV2-ND4 (GS010)	GenSight Biologics	III LTFU	03406104
**Neovascular/age-related macular degeneration**	AAV2-sFLt1	Lions Eye Institute, Perth, Western Australia	I–II, completed	01494805
	AAV2-sFLT01	Genzyme (Sanofi)	I, completed	01024998
	AAV-AntiVEGF	Regenxbio Inc.	I–II	03066258
	AAV-CAG-sCD59	Hemera Biosciences	I	03144999
	AAV7m8-aflibercept	Adverum Biotechnologies, Inc.	I	03748784
	AAV2-CAG-sCD59	Hemera Biosciences	I	03585556
	LV (Retinostat)	Oxford BioMedica	I, completed	01301443
	LV (Retinostat)	Oxford BioMedica	I, LTFU	01678872
	AdGVPEDF.11D	GenVec	I, completed	00109499
**Retinitis pigmentosa**	AAV-ChR2	Allergan	I–II	02556736
	AAV2-VMD2-hMERTK	King Khaled Eye Specialist Hospital	I	01482195
	AAV2.7 m8-CAG-ChrimsonR-tdTomato	GenSight Biologics	I–II	03326336
	AAV8-hRLBP1	Novartis Pharmaceuticals	I–II	03374657
	AAV5-hPDE6B	Horama S.A.	I–II	03328130
**Stargardt disease**	LV (SAR422459)	Sanofi	I–II	01367444
	LV (SAR422459)	Sanofi	I–II (LTFU)	01736592
**Usher syndrome type 1B**	LV (SAR421869)	Sanofi	I–II	01505062
	LV (SAR421869)	Sanofi	I–II (LTFU)	02065011
**Usher syndrome type 2A**	QR-421a	ProQR Therapeutics	I–II	03780257
**X-linked retinitis pigmentosa**	AAV8-RPGR	Nightstar Therapeutics	I–II–III	03116113
	AAV5-hRKp-RPGR	MeiraGTx UK II Ltd	I–II	03252847
	AAV2tYF-GRK1-RPGR	Applied Genetic Technologies Corp	I–II	03316560
**X-linked retinoschisis**	AAV8-scRS/IRBP-hRS	National Eye Institute (NEI)	I–II	02317887
	AAV2tYF-CB-hRS1	Applied Genetic Technologies Corp	I–II	02416622

LTFU: long-term follow-up.

The most successful example of ocular gene therapy to date is represented by subretinal administration of AAV for treatment of LCA type 2 (LCA2). LCA2 is inherited as autosomal recessive and is caused by bi-allelic mutations in *RPE65*, which encodes an essential enzyme of the visual cycle (30). When subretinal delivery of an AAV2 vector encoding for *RPE65* was found to restore vision in the large dog model of LCA2, three different clinical trials involving AAV2-based subretinal delivery of *RPE65* in LCA2 patients were initiated. Despite differences in vector and study design, all trials showed that AAV-mediated gene therapy was safe and effective (31–34), although in two of them a decline in vision was reported several years after vector administration (35,36). More recently, Spark Therapeutics launched an advanced phase III clinical trial, in which bilateral subretinal administrations of AAV2-*RPE65* in LCA2 patients as young as 4 years old confirmed the safety and efficacy profile of the therapy (37), and provided the necessary data to support the market authorization granted initially by the Food and Drug Administration (38), and then by the European Medicinal Agency. Thus, AAV2-*RPE65* (voretigene neparvovec, trade name: Luxturna) represents the first gene therapy product for an ocular disease approved in both US and Europe.

LCA2 is an ideal target for gene therapy because *RPE65* encodes an enzyme expressed in the RPE layer, which is better targeted by AAV vectors than PRs (12); in addition, *RPE65* deficiency causes severe hypovision with a reasonably preserved retinal structure for 2–3 decades (39), which gives gene therapy the opportunity to restore visual function rather than preventing photoreceptor loss, as in many other purely degenerative IRDs. Indeed, AAV-mediated correction of retinitis pigmentosa (RP) due to mutations in *MERTK* (RP38), which like *RPE65* is expressed in the RPE, has also been relied to the clinic after extensive pre-clinical studies. However, only one of six patients showed stable improvements of vision up to 2 years post-injection, while two additional patients showed only transient improvements associated with the treatment (40). This low degree of efficacy might be explained by the degenerative nature of RP38.

Thus, replicating the success of Luxturna will not be an easy task, especially considering that most IRDs require gene transfer to the challenging PR layer. A condition in which there is defective visual function in the presence of preserved retinal structure is achromatopsia, a group of IRDs inherited as autosomal recessive and characterized by poor visual acuity, photophobia, congenital nystagmus and color blindness, as result of defective cone function. Several AAV-based gene therapy trials are targeting achromatopsia (Table 2) and initial safety data from one of them have been recently reported (Michalakis *et al.*, OR006, ESGCT/ISSCR/SFTCG collaborative congress 2018). Other PR diseases for which AAV is at the initial phases of clinical development after successful testing in animals are RP40 due to *PDE6B* mutations and X-linked RP3 due to *RPGR* mutations (Table 2).

AAV vectors have also proven effective in conditions affecting multiple retinal tissues, such as choroideremia (CHM) and X-linked retinoschisis (XLRS), both inherited as X-linked recessive. CHM involves degeneration of choroid, PRs and RPE (41). In 2011, the first phase I–II clinical trial with AAV2 for treating CHM was initiated (NCT01461213) and confirmed that subretinal administration of AAV2 is well tolerated (42). Encouragingly, long-term follow-up of treated patients has later shown small but sustained improvements in visual acuity, up to 5 years post-injection in the longest follow-up (43). Additional trials have confirmed the safety of AAV2 gene therapy for CHM with limited improvements of vision (44,45). In 2018, Nightstar Therapeutics launched a phase III clinical trial (NCT03496012, Table 2), which is expected to enroll 140 CHM patients across several clinical sites to confirm and define the encouraging efficacy results from the previous trials. The clinical results have been less encouraging for XLRS caused by mutations in *RS1* that encodes retinoschisin, a protein involved in the structural organization of the retina. *RS1* is expressed in several retinal cell types, including PRs, ganglion cells, amacrine cells, Müller cells and bipolar cells. The fragile nature of the XLRS retina, characterized by the disruption of the retinal structure and the propensity for developing fluid-filled cysts, favors pan-retinal transduction and rescue following AAV intravitreal administration in mice (46,47,48). The first in-human trial (NCT02317887) testing intravitreal AAV8 administrations in XLRS patients showed no significant gain or loss of visual acuity compared to baseline (49) and that the closure of the typical retinal cysts observed in one participant reopened during the course of ocular inflammation. An additional trial (NCT02416622, Table 2) using an AAV2 tyrosine mutant vector, which transduces the retina from the vitreous with higher efficiency than AAV8 (20), has been recently initiated and the results have not been published yet.

Intravitreal administrations of AAV target efficiently retinal ganglion cells (RGC), which are the target for gene therapy of the severe Leber hereditary optic neuropathy (LHON). LHON is caused by mutations in several genes encoding subunits of the mitochondrial respiratory NADH-ubiquinone oxidoreductase complex in RGC (50). Approximately half of LHON cases are due to mutations in *ND4*, which encodes the NADH dehydrogenase subunit 4. Development of gene therapies for LHON has been limited by the need to deliver therapies to mitochondria (51). To mediate efficient delivery of *ND4* into RGC mitochondria, an N-terminal mitochondrial targeting sequence (52) or both mitochondrial targeting signals and 3’UTRs from nuclear genes whose mRNA has been found to localize to the mitochondrial surface (53) have been successfully used (12,28,54), and further developed into ongoing clinical trials (Table 2). The results published so far have shown that intravitreal administration of AAV2-*ND4* is overall safe, well tolerated and to some extent effective (55). However, improvements in controlateral untreated eyes were also found in some patients (56,57) for which an explanation is still lacking.

### Large gene delivery to the retina

One of the challenges to the broad application of gene therapy with AAV vectors, the only to date able to efficiently transduce PRs, is their packaging capacity limited to approximately 4.7 kb (Table 1) since several IRDs are due to mutations in genes with a larger coding sequence (CDS). This has so far limited the development of valid therapeutic approaches for recessive conditions like (i) Stargardt disease (STGD1), the most common form of inherited macular degeneration, caused by mutations in *ABCA4*; (ii) Usher syndrome type 1B (USH1B), the most severe form of inherited RP and deafness caused by mutations in *MYO7A*; (iii) or LCA type 10, due to mutations in *CEP290*. Thus, significant efforts have been directed towards both the development of AAV-based platforms for large gene delivery and the identification of vectors with higher capacity than AAV, i.e. LV and non-viral vectors, which are able to transduce PRs.

The development of EIAV LV has raised hopes for overcoming the limited PR transduction levels observed with other LV, when adult macaque PR transduction was reported after subretinal administration of this vector (58). This has been the basis for launching in 2011–2012, two Phase I–II clinical trials using EIAV-LV to deliver the large *ABCA4* and *MYO7A* genes to the retina of STGD1 and USH1B patients (NCT01367444 and NCT01505062, Table 2), respectively. The results from these trials have not been published yet, but will be crucial to define the applicability of LV for gene therapy of IRDs due to mutations in large genes expressed in PRs.

To date, the most relevant example of non-viral mediated delivery of a large gene to adult PRs has been obtained using CK30PEG-nanoparticles. They consist of a DNA molecule compacted by polyethylene glycol (PEG)-substituted 30-mer lysine peptides (59). CK30PEG-nanoparticles were found to be non-toxic and non-immunogenic in humans and, most importantly, showed a transgene capacity up to 14 kb (60,61). Interestingly, CK30PEG-nanoparticles have been tested for the delivery of *ABCA4* to the STGD1 mouse retina, resulting in long-term transgene expression as well as structural and functional improvements of the mouse phenotype (61). However, clinical evaluation of this non-viral platform has not started yet.

In parallel, others and us have provided proof-of-concept that large transgenes can be delivered via AAV by splitting them into two separate vectors (dual AAV), which upon co-infection of the same cell reconstitute the expression of a full-length gene via intermolecular recombination between the two AAV vector genomes (62). Interestingly, the levels of protein expression mediated by dual AAV were found to improve the retinal phenotype of mouse models of both STGD1 and USH1B (63–65). Based on these promising results, a phase I–II clinical trial testing the safety and efficacy of dual AAV in the retina of USH1B patients is being planned (https://cordis.europa.eu/project/rcn/212674_it.html), which should define if the levels of expression achieved with dual AAV vectors are therapeutically relevant in humans. More recently, AAV transfer capacity has been expanded up to 14 kb by adding a third vector to the dual system (triple AAV), although this occurred at the expense of efficiency (66). However, the levels of transduction achieved in the retina of a mouse model of Alstrom syndrome with triple AAV led to only modest and transient improvement of the phenotype (66). We and others have shown that the retina represents a favorable environment to develop dual AAV vector-based gene therapy approaches since co-transduction by multiple AAV vectors is quite efficient in the small subretinal space (63,67), especially of large animals (68). Yet, the studies performed so far have shown that none of the dual AAV approaches match the levels of expression achieved with a single AAV vector (63,68,69) and this might be limiting for specific applications. As an alternative platform to overcome AAV limited cargo capacity, we have recently tested intein-mediated protein trans-splicing, which seamlessly reconstitute large proteins from shorter precursor polypeptides (separately encoded by two or more independent AAV vectors) that carry split-inteins at their extremities (70). Interestingly, we found that split-inteins reconstitute large proteins in the retina of small and large animal models and in human retinal organoids, at levels that largely exceed those achieved via dual AAV and match those of a single AAV vector in the large pig retina (Tornabene *et al.*, Sci Transl Med, 2019, in press). The levels of protein reconstitution were sufficient to improve the retinal phenotype of the STGD1 and LCA10 animal models (Tornabene *et al.*, Sci Transl Med, 2019, in press). Defining the safety of this approach will be instrumental for moving AAV intein-mediated protein trans-splicing towards clinical testing.

### Genome editing as a therapeutic option for retinal diseases

IRDs due to gain-of-function mutations are more challenging to tackle by gene therapy since they require silencing/knock-down of the mutant allele. Dominant forms of RP are a major cause of blindness, accounting for more than 30% of RP patients with a recognizable pattern of inheritance (71). Mutations in the rhodopsin gene (*RHO*), several of which are frequent and cause a toxic gain-of-function, are the most common cause of autosomal dominant RP (72). The approaches explored to date to silence *RHO* have been based on either RNA knock-down/suppression or transcriptional repression (72,73). Both allele-specific (i.e. directed to a specific mutation) and allele-independent (directed to both wild-type and mutant *RHO* alleles, followed by addition of a CDS resistant to silencing) have been developed with variable degrees of efficiency (72,73).

In the past decade, the advancement of genome editing, which allows to precisely modify a specific locus, has provided the field with the potential to directly repair mutations underlying human diseases (54,74), in particular those inherited as dominant due to gain-of-function mutations.

Engineered nucleases have been developed for genome editing, and include meganucleases, zinc-finger nucleases, transcription activator-like effector nucleases or clustered regularly interspaced short palindromic repeats (CRISPR)/Cas nucleases (75,76). All allow to precisely induce double-strand breaks at specific genomic loci, which are then repaired by either non-homologous end joining, potentially knocking out mutant gain-of-function alleles, or by homologous recombination, that allows precise correction of the mutation using a donor DNA template (76). Although Cas proteins are derived from bacteria, hence their potential immunogenicity could be a drawback, and their large CDS somehow limit their delivery through AAV vectors, the simplicity of their design, as well as the robustness of editing, have made them the most popular genome editing tool for both research and therapeutic applications, including in the retina (76). Gene correction, which is achieved via homologous recombination, a DNA repair pathway that is inactive in terminally-differentiated neurons, will hardly be efficient in the retina. Several studies have thus investigated CRISPR/Cas9 to knock-out mutant *RHO* alleles in rodent models via non-homologous end joining, with variable degrees of efficacy and specificity (76,77). However, developing allele-specific approaches would be highly expensive and time-consuming. An alternative strategy that exploits non-homologous end joining integrates a donor DNA at a specific locus using CRISPR/Cas9 (78). This homology-independent targeted integration has the potential to provide an allele-independent solution to dominant RP. One of the major concerns associated with the use of CRISPR/Cas9 is the possibility of inducing unintended off-target mutations in the genome; this issue is particularly important in post-mitotic tissues, as the PRs, where AAV-mediated Cas9 expression will persist for long term after a single subretinal injection, thereby increasing the potential for off-target effects. To overcome this issue, self-limiting CRISPR/Cas9 systems as well as delivery of CRISPR/Cas9 ribonucleoproteins consisting of a purified Cas9 protein and a sgRNA are being explored, to achieve transient expression of CRISPR components (76). Also, new variants or orthologues of Cas9 nucleases with higher fidelity have been developed (76). Moreover, ‘dead’ CRISPR/Cas9 variants that retain DNA binding but not cutting activity are being investigated for transcriptional and epigenetic control of DNA expression (76).

In addition to dominant conditions, genome editing may be applicable to recessive diseases due to mutations in genes that are either too large to be packaged into AAV vectors, or toxic if overexpressed from heterologous promoters (79,80). Indeed, CRISPR/Cas9 has been recently tested to correct the frequent IVS26 CEP290 mutation thus abolishing this deleterious *de novo* splice donor site (81,82) . Clinical testing of this strategy is planned for the near future (NCT03872479, Table 2).

### Treating retinal diseases independently of the causative gene

More than 260 genes have been associated with retinal diseases (https://sph.uth.edu/retnet/), which poses a major challenge when developing therapeutic approaches that are gene-specific. For this reason, the identification of mutation-independent strategies has been a longstanding goal of the field. These include neurotrophic molecules delivered via viral vectors to the retina [for a review see (12,77,83)], which have shown the potential to prolong PRs cell survival, although in some cases at the expense of PR function (84). However, none of these strategies has shown significant efficacy in humans (85).

An alternative mutation-independent strategy is represented by optogenetics, which is also particularly suited for advanced cases of IRDs, where no viable PRs are present. In these cases, delivery to bipolar or RGCs of either light-sensitive microbial opsins (86,87), or opsins native to the human eye, as rhodopsin (88) and melanopsin (89), is being explored to activate the retinal circuits downstream of potentially dysfunctional or absent PRs (90). However, either the low light sensitivity of microbial opsins, which requires abnormally high light intensities for activation, or the slow kinetics of melanopsin appears as limitations. Notably, the vertebrate medium wavelength cone opsin (MW-opsin) has been recently shown to provide the speed, sensitivity and adaptation needed to restore patterned vision upon delivery to ganglion cells in the degenerated rd1 mouse retina (91). Another key point for development of effective optogenetics is to restore complex visual responses. Indeed, optogenetics that target RGC, which are the last set of neurons in the retinal network, bypass the stimulus propagation from the inner retinal circuitry. Targeting the outermost surviving retinal layers would instead allow greater levels of signal processing. This can be a challenging task until vectors that are able to efficiently penetrate the retina from the vitreous side are identified, unless effective long-term transduction of bipolar and horizontal cells through subretinal injections is achieved, as it has recently been suggested (92).

## Conclusions

After years of extensive preclinical investigation, research on retinal gene therapy has entered a productive translational phase starting with the first ocular gene therapy product with market approval, and vectors for two additional diseases (CHM and LHON) that are in Phase III of testing. However, some trials have highlighted that preclinical data do not strictly correlate to findings in humans, where in some cases limited or transient improvements were found despite the very promising data obtained in animal models. Whether this is due to a limitation in the ability of the models to either phenocopy the human conditions (36,93) or reflect the levels of vector transduction then obtained in the human retina remains to be understood. Combination of research on the NHP retina, which closely resembles the human, and on human 3D retinal organoids (94) may help improve the level of prediction of pre-clinical studies.

Substantial innovations to the range of technology platforms used for gene therapy have been introduced, which allow to effectively deliver large genes or to knock-out deleterious mutations in animal models. Future clinical testing will ultimately define if these promising pre-clinical results hold true in patients.

In the case of advanced retinal degeneration, therapies based on stem cells, whether genetically-modified or not, will represent a more feasible alternative to *in vivo* gene delivery for vision restoration (95–97). Yet, thus far, RPE cells have been generated *in vitro* more easily and reproducibly than PRs, whose first-in-human may be however at the horizon.

In conclusion, with the advancements made by gene therapy in the past decades, and those that will come in the short term, identification of treatment options for a number of blinding diseases seems now at reach.

## Funding

European Research Council (ERC) (grant numbers 694323 ‘EYEGET’; 754848 ‘USHTHER’; 825825 ‘UPGRADE’ to A.A.); Telethon Foundation (grant TGM16MT1); University of Naples Federico II under STAR Program (to I.T.).

## Conflict of Interest statement

None declared.
